# Association between human herpes simplex virus and periodontitis: results from the continuous National Health and Nutrition Examination Survey 2009–2014

**DOI:** 10.1186/s12903-023-03416-x

**Published:** 2023-09-19

**Authors:** Yansong Song, Na Liu, Lijie Gao, Dan Yang, Jiaxin Liu, Liang Xie, Hongxia Dan, Qianming Chen

**Affiliations:** 1 State Key Laboratory of Oral Diseases & National Center for Stomatology & National Clinical Research Center for Oral Diseases & Research Unit of Oral Carcinogenesis and Management, Chinese Academy of Medical Sciences, West China Hospital of Stomatology, Sichuan University, Chengdu, 610041 Sichuan China; 2 Department of Periodontics, Guangdong Engineering Research Center of Oral Restoration and Reconstruction, Guangzhou Key Laboratory of Basic and Applied Research of Oral Regenerative Medicine, Affiliated Stomatology Hospital of Guangzhou Medical University, Guangzhou, 510182 China; 3 Department of Neurology, West China Hospital, Sichuan University Chengdu, Sichuan, 610041 People’s Republic of China

**Keywords:** Human herpes simplex virus, Periodontitis, National Health and Nutrition Examination Survey, Risk assessment

## Abstract

**Background:**

Periodontitis is a common chronic oral disease which seriously affects people's quality of life. Although human herpes simplex virus (HSV) is also found in periodontal lesions, the association between HSV infection and periodontitis is unclear.

**Methods:**

The National Health and Nutrition Examination Survey (NHANES) data for 2009–2010, 2011–2012 and 2013–2014 was combined, and the association between HSV infection and periodontitis in the general population and particular subgroups was investigated through weighted multi-logistic analyses.

**Results:**

There were 4,733 participants aged 30–50 years old with clinically assessed periodontitis concurrent with HSV infection. In general analysis, after adjusted for covariates, both HSV-1 (OR = 1.09, *P* < 0.001) and HSV-2 (OR = 1.06, *P* = 0.030) infection was significantly associated with periodontitis. In subgroup analyses, compared with patients without HSV infection, patients with HSV-1( +) & HSV-2( +) and HSV-1( +) & HSV-2(-) infection showed higher risk of periodontitis in all subgroups (OR = 1.15, OR = 1.09, *P* < 0.001), while patients with HSV-1(-) & HSV-2( +) infection showed higher risk of and periodontitis only in the subgroup of people aged 40–50 years (OR = 1.10, *P* = 0.032) and the Mexican–American subgroup (OR = 1.35, *P* = 0.042). When only severe periodontitis is considered, HSV infection was associated with periodontitis, no matter the patient was infected with either of the virus or both.

**Conclusions:**

HSV-1 infection was significantly associated with periodontitis and severe periodontitis, while HSV-2 infection was associated with severe periodontitis, and periodontitis in 40–50-year-olds and Mexican-Americans.

## Background

The human herpes simplex virus (HSV), which includes HSV-1 and HSV-2, belongs to the alpha (α) subfamily of the *Herpesviridae* family [1]. Herpesvirus virions include a DNA molecule, an icosahedral capsid, a proteinaceous tegument, and an envelope deriving from host cell membrane and are about 0.25 μm in diameter [2]. α-herpesviruses can produce rapid lysis of the infected cells in sensory ganglia. HSV-1 infection can cause corneal keratitis and/or cold sores while infection from HSV-2 mainly causes genital disease. In some cases, HSV-1 can cause genital herpes, and HSV-2 may cause oral or facial herpes. Herpesviruses have several mechanisms to evade host immunity, for example, encoding immune-evasive glycoproteins, avoiding T-cell immune surveillance, and interfering natural killer cell function [1, 3].

Periodontitis is a complicated chronic oral disease that can eventually lead to the rupture of periodontal ligaments, alveolar bone destruction, and tooth loss [4]. The pathogenic factors of periodontitis involve a biofilm composed of bacteria and viruses, the response of host's immune system to it, and genetic factors [5–8]. The peak of periodontitis prevalence occurs between 35 to 40 years [9]. In 2017, 796 million individuals worldwide had severe periodontitis [10]. In the United States, 42.2% of adults (≥ 30 years old) had periodontitis, 7.8% of them with a severe form [11]. A nonlinear dose–response association between the incidence of periodontitis and vitamin E was found. Within reasonable limits, supplemental intake helped reduce the prevalence of periodontitis, while excessive intake did not help significantly and might even increase the risk [12]. Meta-analyses confirmed that periodontitis was associated with white cell lineages. Severe periodontitis was associated with higher white blood cell counts when compared with controls [13]. Furthermore, the association of periodontitis with ≥ 57 different systemic diseases have been studied, and positive association has been found between periodontitis and systemic diseases such as diabetes types 1 and 2, atherogenesis and ACVD, renal dysfunction, premature and dysmature born babies and other pregnancy complications [14], partly due to the continuous release of inflammatory factors and other immune biomarkers [15]. On the other hand, periodontal herpesviruses could infect distant organs via the systemic circulation [16], and an HSV infection may promote some cardiovascular diseases caused by periodontal pathogens [17].

HSV-1 virus has been found in the periodontal and pulp tissues of patients with periodontitis and necrotic pulp [18]. After the discovery of HSV in periodontitis, its potential pathogenic role in this disease has been investigated [19, 20]. Several studies had investigated the association between HSV infection and periodontitis, the results were quite controversial [21–25]. However, the sample sizes of most studies were small, HSV-1 and HSV-2 infection were not always simultaneously tested, and the confounding factors were not adjusted in some studies. Therefore, the conclusions obtained may be inaccurate.

The aim of the current study was to get a clearer relationship between periodontitis and both types of HSV using a larger sample size, and stratified analysis and multifactor logistics regression analysis to eliminate the effect of confounding factors.

## Methods

### Study design and population

NHANES was a cross-sectional survey applying stratified, multistage, probability sampling design that started early in the 60 s. The National Center for Health Statistics conducted this project, aiming at evaluating the health and nutritional status of American residents. Since 1999, the survey data, focusing on a variety of nutrition and health measurements, has been released every two years. The three survey cycles for the periodontal examination with six sites of full tooth from 2009–2014 were included. Participants who were age 30 or older prior to the periodontal examination without health conditions requiring antibiotic prophylaxis could be included. Participants who had received a heart transplant, an artificial heart valve, congenital heart disease (excluding mitral valve prolapse), or a history of bacterial endocarditis were excluded from the periodontal examination. Next, participants without full periodontal examination, and those patients who did not have HSV antibody test results or were not suitable for HSV antibody examination were excluded.

### Definition of periodontitis

Between 2009–2014, the NHANES periodontal examination was performed in participants > 30 years old. Trained dental hygienists in the United States (US) examined periodontal pockets, recession, and loss of attachment in suitable participants. We classified the grade and severity of periodontitis according to the following criteria [26]: no periodontitis: no evidence of mild, moderate, and severe periodontitis; mild periodontitis ≥ 2 interproximal sites with attachment loss (AL) ≥ 3 mm and < 4 mm and ≥ 2 interproximal sites with probing depth (PD) ≥ 4 mm on different teeth, or one site with PD ≥ 5 mm; moderate periodontitis: ≥ 2 interproximal sites with AL ≥ 4 mm and < 6 mm not on the same tooth or ≥ 2 interproximal sites with PD ≥ 5 mm on different teeth; and severe periodontitis: ≥ 2 interproximal sites with AL ≥ 6 mm on different teeth and ≥ 1 interproximal sites with PD ≥ 5 mm.

### HSV infection

Serum HSV-1 and HSV-2 antibodies were detected in 2009–2014 NHANES participants aged 14–49 years old, and 18–49 years old, respectively. Specific glycoprotein G (gG-1and gG-2) were used to detect HSV-1 and HSV-2 [27]. These glycoproteins have been purified by monoclonal antibodies and affinity chromatography and tested by solid-phase enzymatic immunodot assays [28]. According to HSV antibody detection status, HSV infection condition was categorized as follows: HSV-1 ( +), HSV-2 ( +), HSV-1 ( +) & HSV-2 (-), HSV-1(-) & HSV-2 ( +), HSV-1 ( +) & HSV-2 ( +), HSV-1(-) & HSV-2 (-).

### Covariates

Demographic variables from the demographic questionnaire, such as age, sex, race/ethnicity, education, marital status, and poverty-to-income ratio (PIR, which is a ratio of family income to poverty threshold), were included as covariates. Age was calculated as the time from birth to interview. Sex was self-reported as either male or female. Race/ethnicity was classified as non-Hispanic White, non-Hispanic Black, Mexican American, and other Hispanic. Education levels were collected and classified into five levels (less than 9th grade, 9-11th grade, high school graduate, some college, college graduate or above) with consideration of practical interpretation and sample size within levels. Marital status was classified as married, living with partner, widowed, divorced, separated, never married. We cut PIR into a categorical variable at the value of 1 and 3. Participants with < 100 cigarettes smoked in a lifetime were defined as never smoking. Previous smoking was defined as having smoked > 100 cigarettes in their lifetime but no longer smoking. Current smoking was defined as sometimes or everyday smoking > 100 cigarettes in lifetime. Alcohol consumption was defined as yes, if participants had > 10 drinks in their lifetime. Physical activity was treated as yes, when participants performed vigorous work activity. Diabetes was determined by the doctor telling the patient whether they had diabetes (yes, borderline, no). Body mass index (BMI) was calculated as weight/height2 (kg/m2) and classified into < 25, ≥ 25 and < 30, and ≥ 30 kg/m^2^ subgroups. Vitamin E intake from daily diet was collected from the first day total nutrients intake questionnaire. White blood cell count and serum cholesterol as covariates were collected from the NHANES laboratory data. Serum cholesterol was measured by the Roche Modular P chemistry analyzer. White blood cell counts were measured by the Beckman Coulter MAXM instrument at the NHANES Mobile Examination Centers (MECs).

### Statistical analyses

Continuous variables are demonstrated as mean ± standard error (SE) and categorical variables as unweighted number (weighted percentage) in descriptive analysis. The basic characteristic differences among periodontitis subgroups for continuous and categorical variables were tested by the one‐way analysis of variance and chi‐squared, respectively. Weighted univariate and multivariate logistic regression analyses were applied to study the association of different HSV infection conditions and periodontitis. Age, sex, education, race/ethnicity, marital status, PIR, smoking status, alcohol consumption, physical activity, BMI, diabetes, vitamin E intake, white blood cell count, and total serum cholesterol were adjusted for. We extracted variables related to demographic characteristics to create subgroups. We further performed stratified analyses to study the association between HSV infection condition and periodontitis in various subgroups. To ensure sample sizes for the different subgroups in the stratified analysis, we dichotomized the age variable (age ≥ 30, < 40 years; age ≥ 40, < 50 years). We combined the 3-year weight to perform weighted analyses, as indicated in NHANES guidelines [29]. *P* < 0.05 was treated as statistically significant. Since the number of missing data in this study was small and the valid data was sufficient, the deletion method was adopted to deal with the missing data. R 4.0.1was used to conduct all analyses. (http://www.R-project.org).

## Results

### Basic characteristics of analyzed participants

**Fig. 1:**
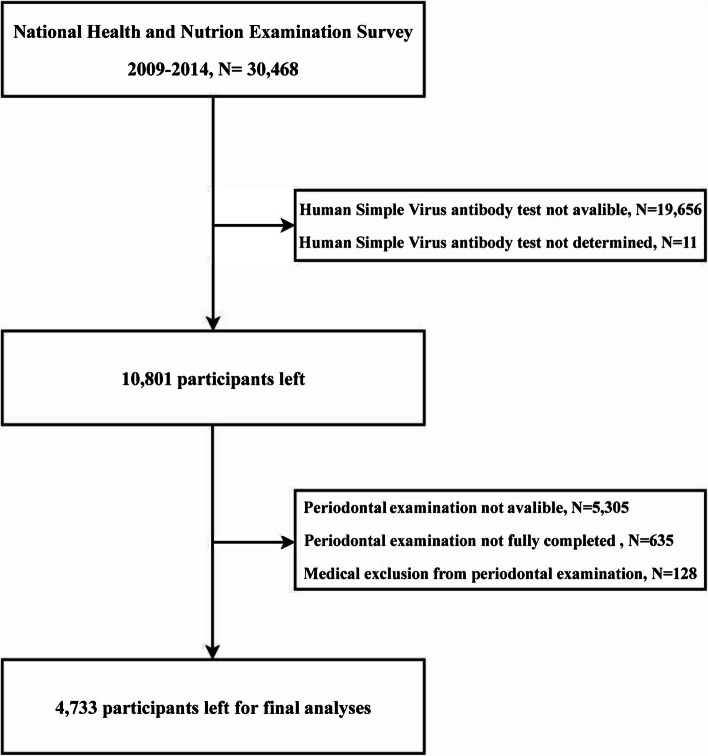
Flowchart of study participants from NHANES 2009–2014 survey

The 2009–2014 NHANES included responses from 30,468 American residents. After excluding participants with human HSV antibody test not available (*N* = 19,656) or undetermined (*N* = 11), those without available periodontal examination (*N* = 5,305), medical exclusion of periodontal examination (*N* = 128) or incomplete periodontal examination (*N* = 635), 4,733 participants aged between 30–50 years were included for final analyses (Fig. 1).

As presented in Table 1, the ratio of patients of different races, education levels, marital status, smoking habits, and physical activities were all different between periodontitis and non-periodontitis groups (*P* < 0.001). The percentages of Non-Hispanic White race (52.3%), college graduate or above (38.4%), married participants (59.3%) in non-periodontitis subgroups were much higher than in periodontitis group (*P* < 0.001). PIR distribution was also significantly different (*P* < 0.001). There were significantly more participants with periodontitis who were currently smoking (33.5%) and performed physical activity (42.7%) than non-periodontitis participants (*P* < 0.001). Low Vitamin E intake was associated with periodontitis (*P* = 0.003). The mean BMI, white blood cell count, and total serum cholesterol were prominently higher in the periodontitis subgroup than in the non-periodontitis subgroup (*P* < 0.001).

**Table 1:** Characteristics of study participants grouped by periodontitis condition

Variables	Total population^a^ (*n* = 4,733)	Non-periodontitis^a^ (*n* = 2,702)	Periodontitis^a^ (*n* = 2,031)	*P* Value
Age, Mean ± SE	39.5 ± 0.1	38.8 ± 0.2	40.5 ± 0.2	< 0.001
Men, n (%)	2303 (51.3)	1087(43.2)	1216(63.2)	< 0.001
Race, n (%)				< 0.001
Mexican American	746 (15.2)	311(11.1)	435(21.3)	
Other Hispanic	475 (8.7)	254(8.5)	221(9.1)	
Non-Hispanic White	2011 (47.0)	1337(52.3)	674(39.3)	
Non-Hispanic Black	844 (17.0)	414(15.3)	430(19.4)	
Other Race	657 (11.2)	386(12.9)	271(10.9)	
Education, n (%)				< 0.001
Less than 9th grade	343 (6.4)	114(3.7)	229(10.3)	
9-11th grade	654 (14.5)	270(10.0)	384(21.2)	
High school graduate	992 (21.4)	447(17.0)	545(28.1)	
Some college	1365 (28.2)	823(30.8)	542(24.2)	
College graduate or above	1375 (29.5)	1047(38.4)	328(16.2)	
Marital Status, n (%)				< 0.001
Married	2804 (59.3)	1700(63.4)	1104(53.4)	
Living with partner	43 (1.0)	19(0.9)	24(0.9)	
Widowed	496 (11)	268(8.9)	228(14)	
Divorced	179 (3.4)	76(3)	103(4)	
Separated	732 (15.5)	424(16.4)	308(14.1)	
Never married	478(9.9)	215(7.4)	263(13.7)	
PIR, n (%)				< 0.001
< 1	950 (22.0)	412(15.6)	538(31.6)	
≥ 1 and < 3	1680 (38.5)	862(35.0)	818(43.3)	
≥ 3	1738 (39.5)	1259(49.3)	479(25.0)	
Smoking status, n (%)				< 0.001
Never	2805 (59.5)	1757(66)	1048(49.9)	
Previous	807 (16.8)	482(17.0)	325(16.6)	
Current	1120 (23.7)	463(16.9)	657(33.5)	
Alcohol consumption, n (%)	3318 (78.2)	1912(77.7)	1406(79.2)	0.646
Physical activity, n (%)	1782 (39.6)	958(37.7)	824(42.7)	0.023
BMI, Mean ± SE	29.46 ± 0.2	29.3 ± 0.2	29.7 ± 0.3	0.007
Diabetes, n (%)				0.033
No	4391 (92.2)	2538(93.6)	1853(90.1)	
Borderline	74 (1.6)	41(1.6)	33(1.6)	
Yes	265 (6.2)	120(4.8)	145(8.3)	
Vitamin E intake, Mean ± SE	8.61 ± 0.2	9.1 ± 0.2	7.9 ± 0.2	0.003
White blood cell, Mean ± SE	6.9 ± 0.1	6.8 ± 0.1	7.1 ± 0.1	< 0.001
Total cholesterol, Mean ± SE	5.08 ± 0.0	5.0 ± 0.0	5.2 ± 0.0	< 0.001

Abbreviations *PIR* Poverty to income ratio, *BMI* Body mass index

^a^For category variables unweighted number and weighted percentage were presented

### Prevalence of periodontitis according to HSV infection

**Fig. 2:**
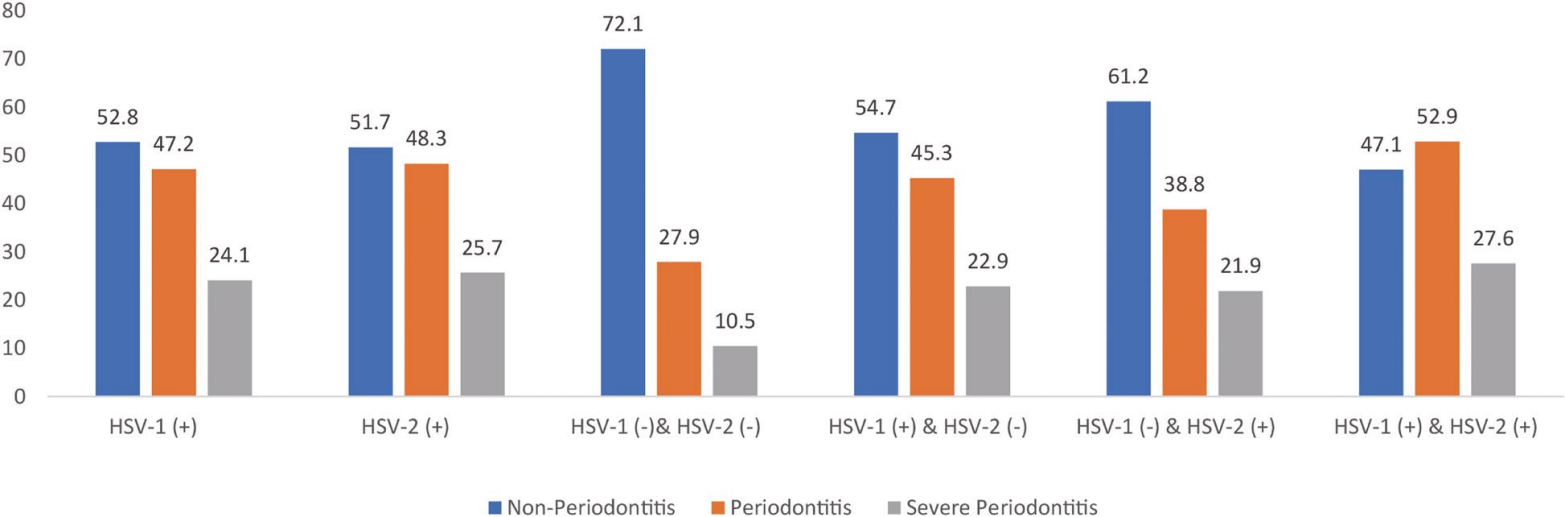
Weighted prevalence of periodontitis and severe periodontitis among HSV infection among NHANES 2009–2014 survey. The variance analysis was performed between periodontitis and non-periodontitis (*P* < 0.001)

Participants were divided into six subgroups according to HSV-1 and HSV-2 infected condition. Figure 2 shows the prevalence of non-periodontitis, periodontitis, and severe periodontitis in these six subgroups. The prevalence of periodontitis (52.9%) and severe periodontitis (27.6%) was highest in the HSV-1 ( +) & HSV-2 ( +) subgroup, followed by the HSV-1 ( +) & HSV-2 (-) subgroup, HSV-1 (-) & HSV-2 ( +) subgroup and HSV-1 (-) & HSV-2 (-) *subgroup.*

### Association between HSV and periodontitis

In the crude model of risk factors for periodontitis, HSV-1 ( +) participants had an 18% higher risk of periodontitis (OR 1.18, 95% CI: 1.13–1.24; *P* < 0.001) than HSV-1 (-) participants. After adjusting for age, sex, race/ethnicity, education, marital status, smoking status, PIR, alcohol consumption, BMI, physical activity, diabetes, vitamin E intake, white blood cell count, and total serum cholesterol, HSV-1 infection was still significantly related to periodontitis (OR 1.09, 95% CI: 1.05–1.14; *P* < 0.001). Similarly, HSV-2 ( +) individuals also had higher risk of periodontitis in both the crude model (OR 1.09, 95%CI: 1.04–1.15, *P* < 0.001) and the adjusted model (OR 1.06, 95% CI: 1.01–1.11; *P* < 0.001). Participants were further divided into different subgroups according to HSV-1 and HSV-2 infection. When HSV-1 (-) & HSV-2 (-) subgroup was set as the reference group, both HSV-1 ( +) & HSV-2 (-) participants and HSV-1 ( +) & HSV-2 ( +) participants showed higher risk of periodontitis in the adjusted model (HSV-1 ( +) & HSV-2(-): OR 1.09, 95% CI: 1.04–1.14, *P* < 0.001; HSV-1 ( +) & HSV-2 ( +): OR 1.15, 95% CI: 1.09–1.22, *P* < 0.001). However, in the adjusted model, the risk of periodontitis was not significantly different between HSV-1 (-) & HSV-2 ( +) group and the reference group (OR 1.05, 95% CI: 0.98–1.14; *P* = 0.140; Table 2).

**Table 2:** Association between HSV infection and periodontitis among participants from NHANES 2009–2014 survey

	Crude Model			Adjusted Model^a^		
	OR	95% CI	*P*	OR	95% CI	*P*
HSV-1 ( +)	1.18	1.13,1.24	< 0.001	1.09	1.05,1.14	< 0.001
HSV-2 ( +)	1.09	1.04,1.15	< 0.001	1.06	1.01,1.11	0.030
HSV Antibody						
HSV-1(-) & HSV-2 (-)	1	Reference		1	Reference	
HSV-1 ( +) & HSV-2 (-)	1.19	1.13,1.25	< 0.001	1.09	1.04,1.14	< 0.001
HSV-1 (-) & HSV2 ( +)	1.11	1.03,1.21	0.01	1.06	0.98,1.14	0.140
HSV-1 ( +) & HSV-2 ( +)	1.28	1.21,1.37	< 0.001	1.15	1.09,1.22	< 0.001

*Abbreviations*: *OR* Odds ratio, *CI* Confidence interval, *HSV* Human Simple Virus

^a^Age, sex, race, educat0ion, marital status, PIR, smoking status, alcohol consumption, physical activity, BMI, diabetes, vitamin E intake, white blood cell count, and total serum cholesterol were adjusted

Table 3 showed the relation between HSV infection and severe periodontitis among young adults. HSV-1 ( +) or HSV-2 ( +) infection was notably associated with a higher prevalence of severe periodontitis (HSV-1 ( +), OR 1.06, 95% CI 1.03–1.09, *P* < 0.001; HSV-2 ( +), OR 1.05, 95% CI 1.01–1.09, *P* = 0.020). When the HSV-1 (-) & HSV-2(-) subgroup was set as the reference group, HSV-1 ( +) & HSV-2(-) participants had a 7% higher risk of severe periodontitis (OR 1.07, 95% CI 1.03–1.10, *P* = 0.020), while the HSV-1 ( +) & HSV-2 ( +) subgroup had a 12% higher risk of severe periodontitis (OR 1.12, 95% CI 1.06–1.18, *P* < 0.001). HSV-1 (-) & HSV-2 ( +) subgroup also had 8% higher risk of severe periodontitis (OR 1.08, 95% CI 1.03–1.13, *P* = 0.049) compared to the reference group.

**Table 3:** Association between HSV infection and severe periodontitis among participants from NHANES 2009–2014 survey

	Crude Model			Adjusted Model^a^		
	OR	95% CI	*P*	OR	95% CI	*P*
HSV-1 Positive	1.11	1.08,1.15	< 0.001	1.06	1.03,1.09	< 0.001
HSV-2 Positive	1.07	1.03,1.11	< 0.001	1.05	1.01,1.09	0.020
HSV Antibody						
HSV-1 (-) & HSV-2 (-)	1	Reference		1	Reference	
HSV-1 ( +) & HSV-2 (-)	1.13	1.10,1.17	< 0.001	1.07	1.03,1.10	< 0.001
HSV-1 (-) & HSV-2 ( +)	1.12	1.05,1.20	0.010	1.08	1.03,1.13	0.049
HSV-1 ( +) & HSV-2 ( +)	1.19	1.13,1.25	< 0.001	1.12	1.06,1.18	< 0.001

*Abbreviations*: *OR* Odds ratio, *CI* Confidence interval, *HSV* Human Simple Virus

^a^Age, sex, race, educat0ion, marital status, PIR, smoking status, alcohol consumption, physical activity, BMI, diabetes, vitamin E intake, white blood cell count, and total serum cholesterol were adjusted

### Subgroup analyses of association between HSV and periodontitis

**Fig. 3:**
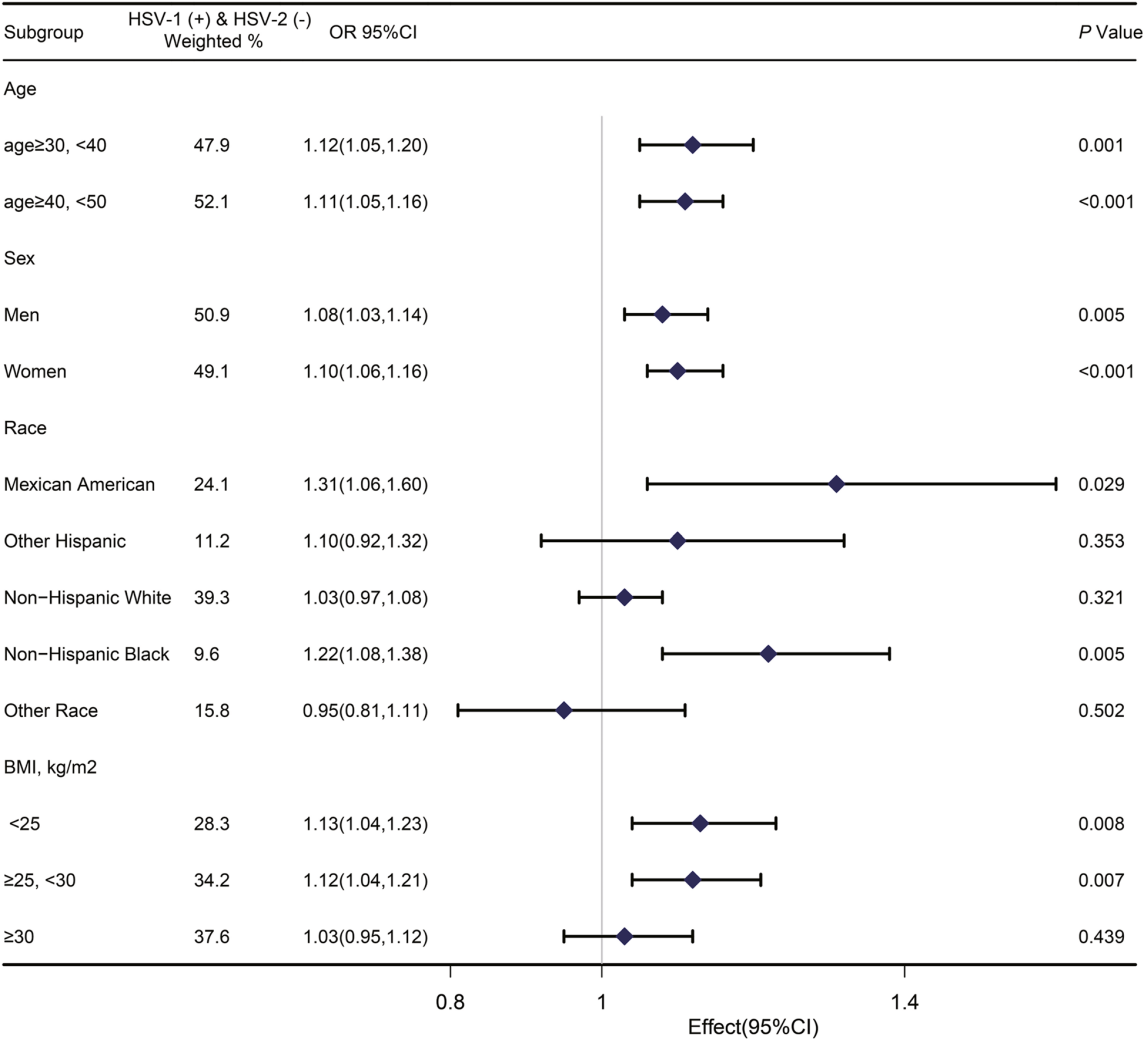
Association between HSV1 positive and HSV2 negative infection and periodontitis among subgroups of participants. Age, sex, race, educat0ion, marital status, PIR, smoking status, alcohol consumption, physical activity, BMI, diabetes, vitamin E intake, white blood cell count, and total serum cholesterol were adjusted except for the stratified factor

**Fig. 4:**
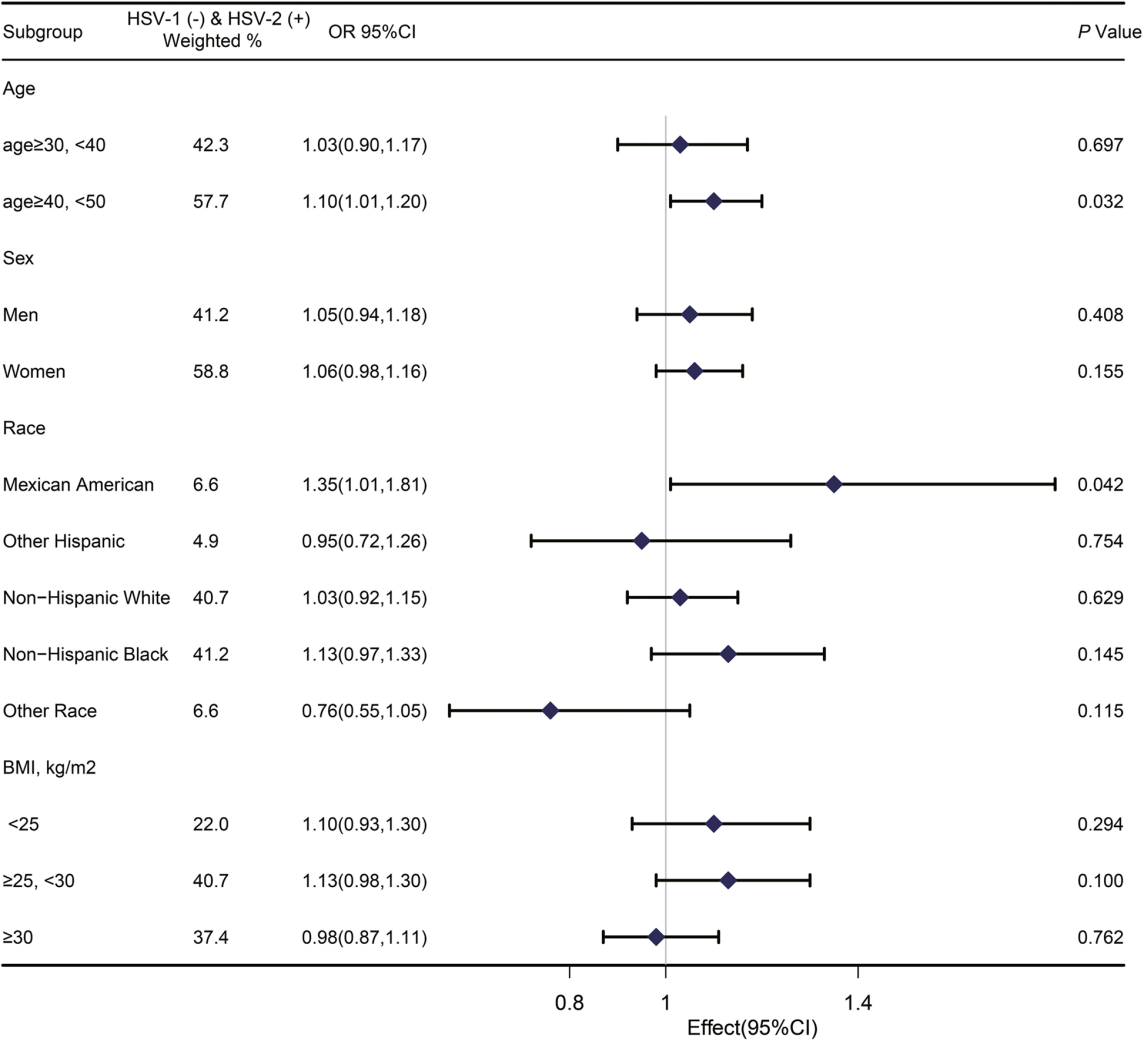
Association between HSV1 negative and HSV2 positive infection and periodontitis among subgroups of participants. Age, sex, race, educat0ion, marital status, PIR, smoking status, alcohol consumption, physical activity, BMI, diabetes, vitamin E intake, white blood cell count, and total serum cholesterol were adjusted except for the stratified factor

In Figs. 3 and 4, HSV-1 (-) & HSV-2 (-) participants were set as the reference group. Figure 3 shows the relation between HSV-1 ( +) & HSV-2 (-) infection and periodontitis in various subgroups. HSV-1 ( +) & HSV-2 (-) infection was associated with higher risk of periodontitis in both age subgroups, both sexes, but only in Mexican–American, Non-Hispanic Black subgroups and subgroups with BMI < 30. However, HSV-1 (-) & HSV-2 ( +) infection was associated with higher risk of periodontitis only in the second age group (age ≥ 40, < 50 years) and the Mexican–American subgroup (Fig. 4).

## Discussion

Development of periodontitis is related with multiple factors. In our analysis of 4,733 US adult samples (30–50 years old), we found that increasing age (especially from 40 years old), smoking, and male sex were associated with the development of periodontitis. All these were recognized risk factors for periodontitis [9, 30]. Participants of Non-Hispanic White race, with a high level of education, a stable emotional life (married participants), and a good income were less likely to develop periodontitis. This was consistent with previous studies showing that higher income and lower stress could help reduce the incidence of periodontitis [31, 32]. Our results showed that high white blood cell count was related to periodontitis. This was consistent with findings of Botelho's recent study, in which a causal relationship between periodontitis and high white blood cell counts was suggested [13]. We also found that participants with increased BMI and high total serum cholesterol were at increased risk of periodontitis, since those factors indicated obesity, that might increase their likelihood to develop diabetes, which is a risk factor for periodontitis [33]. In addition, our results suggested that low vitamin E intake was associated with the development of periodontitis, which is somewhat supported by the findings of Bas et al. in rats that systemic vitamin E supplementation helped treat periodontitis [34].

However, it was found that physical activity but not alcohol consumption was associated with the development of periodontitis. These findings were not in accord with previous findings [35], which might be caused by the different standards used to measure alcohol consumption, and the possibility that the criteria for physical activity could not fully reflect the physical movements of participants.

HSV-1 had been detected in periodontitis [18, 21, 23, 36]. Some saliva factors could enhance the infection of gingival fibroblasts by HSV-1 [37]. However, the association between HSV infection and periodontitis was still quite controversial. A study by Stein et al. reported no association between HSV and periodontitis. But only 65 Germans were included in this study, and only one subtype, HSV-1, was tested [24]. A meta-analysis (2015) suggested an inconclusive association between HSV and periodontitis. Only two articles about HSV infection were included in this meta-analysis, the sample size was only 30. And only HSV and HSV-1 were examined, not HSV-2 [25]. Nevertheless, other studies reported that HSV-1 is associated with periodontitis. In one study, HSV-1 was detected in 24.7% of total 89 patients [21]. In another study, more than 100 patients with HSV-1 or HSV-2 infection were included, and HSV-1 was the most common herpes virus associated with periodontitis. However, adjustment for confounding factors was lacking [22]. And another meta-analysis (2017) suggested that HSV-1 is associated with periodontitis. The relationship between HSV-2 and periodontitis is still controversial. The sample size was just over a dozen patients, and adjustment for confounding factors was not performed in it [23].

HSV infection was associated with higher percentage of periodontitis and severe periodontitis. Previous studies indicated that HSVs may play a role in the dynamic pathogenesis of periodontal disease. HSVs are capable of multiplying in gingival tissue. They may have a direct cytopathic effect on fibroblasts, keratinocytes, endothelial cells and inflammatory cells such as polymorphonuclear cells, lymphocyte, macrophages and bone cells [38]. HSVs may also mediate damage to the host immune response and thus play a role in the pathogenesis of periodontal diseases [39]. HSVs can also be observed to play a role in severe periodontitis in the practice of treatment of severe periodontitis. Some studies reported the use of antivirals to treat the refractory periodontitis [40].

We further subdivided the HSV infection status. According to our findings, HSV-1 (-) & HSV2 ( +) infection was not significantly associated with periodontitis but the HSV-1 ( +) & HSV2 (-) infection was. The possible reasons for this result may be multifactorial. There was a difference in the detection rate of the two HSV subtypes in periodontitis. Studies have reported significantly higher detection rates for HSV-1 than for HSV-2 [41]. On the other hand, the study also claimed that the presence of HSV-1 was positively associated with the presence of *Porphyromonas gingivalis*, *Prevotella intermedia*, *Tannerella forsythia*, and *Campylobacter rectus*. Whereas, the presence of HSV-2 was not associated with the presence of putative pathogenic bacteria in the above-mentioned periodontitis lesions [41].

Our results revealed that HSV-1 infection was notably associated with periodontitis and HSV-2 was associated with periodontitis only in the subgroups of people aged between 40–50 years and the Mexican–Americans. In disagreement with previous studies, HSV-1 was significantly associated with periodontitis [21–23]. In contrast, almost all previous studies have found no relationship between HSV-2 and periodontitis. In our study, we found that HSV-2 was associated with periodontitis in specific subgroups, and that both HSV-1 and HSV-2 were related to the occurrence of severe periodontitis. A reasonable explanation for this phenomenon is that the HSV‐bacteria interaction contributes to the onset of rapid, severe periodontitis. The traditional etiology of periodontitis suggests its formation by calculus and biofilms containing non-specific microorganisms [42]. However, this theory fails to explain the following two phenomena: the low occurrence of periodontitis in people with a biofilm mass and, conversely, the beginning and rapid progression of typical juvenile periodontitis with virtually no detectable calculus and biofilms. In the late 1990s, the role of HSV in the etiology of periodontitis was noted, and it has been assumed that the microorganisms that cause periodontitis are specific [20, 43, 44]. Once infected with HSV, the host becomes a lifetime carrier. The balance between HSV toxicity and the host's anti-HSV defense mechanism may be critical in determining the development of periodontitis. On the other hand, the destruction of oral epithelial barrier by periodontitis may also aggravate the invasion and virulence of HSV. Moreover, saliva from certain populations can increase the susceptibility of gingival fibroblasts to HSV-1 [37]. Similarly, the Mexican subgroup may have a genetic susceptibility to HSV-2, and localized juvenile periodontitis may be due to hormonal induction in adolescence or loss of host immune defense, which leads to the reactivation of latent HSV in the periodontal region [45].

Our results are based on a nationally representative sample of U.S. adults with CVD, and adjusted for a variety of potential confounding factors. However, there are also some limitations in our study. Firstly, due to the observational study design, causality could not be determined. Secondly, covariates relied on self-reporting at baseline, which may reduce accuracy and could not reflect changes over time. Although the current study suggested a relationship of HSV, especially HSV-1 infection with periodontitis, the results should be interpreted cautiously. Whether HSV infection contributes to the development of periodontitis or is just the consequence of periodontal destruction still needs to be explored. Follow-up studies of the development of periodontitis in patients with or without HSV infection, detection of the viral load of HSV in patients with different severity of periodontitis, study of the interactions of HSV and periodontal tissue in cell and animal models may help us to get a clearer understanding of the association of HSV infection and periodontitis.

## Conclusions

Our study showed that HSV-1 infection was significantly related to periodontitis. HSV-2 was associated with periodontitis only in the subgroups of people aged between 40–50 years and the Mexican–Americans. Both HSV-1 and HSV-2 were related to the occurrence of severe periodontitis. The results of this study suggested an association between HSV infection and the development of periodontitis. It is necessary to consider HSV infection in the prevention and management of periodontitis.

## Data Availability

All data analyzed in this study can be downloaded from NHANES. https://wwwn.cdc.gov/nchs/nhanes/Default.aspx

## Abbreviations

HSV: Herpes simplex virus
NHANES: National health and nutrition examination survey
US: The United States
AL: Attachment loss
PD: Probing depth
gG: Glycoprotein G
PIG: Poverty-to-income ratio
BMI: Body mass index
SE: Standard error
OR: Odds ratio
CI: Confidence interval
